# Healthcare professionals’ perceptions on barriers and facilitators to DMARD use in rheumatoid arthritis

**DOI:** 10.1186/s12913-021-07459-0

**Published:** 2022-01-13

**Authors:** M. J. H. Voshaar, B. J. F. van den Bemt, M. A. F. J. van de Laar, A. M. van Dulmen, J. E. Vriezekolk

**Affiliations:** 1 Department of Pharmacy and Department of Research & Innovation, Sint Maartenskliniek, Ubbergen, The Netherlands; 2 Department of Pharmacy, Radboud university medical center, Nijmegen, The Netherlands; 3 Department of Clinical Pharmacy and Toxicology, Maastricht University Medical Center, Maastricht, The Netherlands; 4 University of Twente, P.O box 50,000, 7500 KA Enschede, The Netherlands; 5 Netherlands institute for health services research, Utrecht, The Netherlands; 6 Radboud university medical center, Radboud Institute for Health Sciences, Department of Primary and Community Care, Nijmegen, The Netherlands; 7 Department of Research & Innovation, Sint Maartenskliniek, Ubbergen, The Netherlands

**Keywords:** Healthcare professionals, DMARDs, Barriers, Facilitators, Medication adherence, Rheumatoid arthritis

## Abstract

**Background:**

Disease-modifying anti-rheumatic drugs (DMARDs) are the cornerstone of rheumatoid arthritis (RA) treatment. However, the full benefits of DMARDs are often not realized because many patients are sub-optimally adherent to their medication. In order to optimize adherence, it is essential that healthcare professionals (HCPs) understand patients’ barriers and facilitators for medication use. Insight in these barriers and facilitators may foster the dialogue about adequate medication use between HCPs and patients. What HCPs perceive as barriers and facilitators has, so far, scarcely been investigated. This study aimed to identify the perceptions of HCPs on patients’ barriers and facilitators that might influence their adherence.

**Methods:**

This qualitative study was performed using semi structured in-depth interviews with HCPs. An interview guide was used, based on an adjusted version of the Theoretical Domains Framework (TDF). Thematic analysis was conducted to identify factors that influence barriers and facilitators to DMARD use according to HCPs.

**Results:**

Fifteen HCPs (5 rheumatologists, 5 nurses and 5 pharmacists) were interviewed. They mentioned a variety of factors that, according to their perceptions, influence DMARD adherence in patients with RA. Besides therapy-related factors, such as (onset of) medication effectiveness and side-effects, most variation was found within patient-related factors and reflected patients’ beliefs, ways of coping, and (self-management) skills toward medication and their condition. In addition, factors related to the condition (e.g., level of disease activity), healthcare team and system (e.g., trust in HCP), and social and economic context (e.g. support, work shifts) were reported.

**Conclusions:**

This study provided insights in HCPs’ perceptions of the barriers and facilitators to DMARD use patients with RA. Most factors that were mentioned were patient-related and potentially modifiable. When physicians understand patients’ perceptions on medication use, adherence to DMARDs can probably be optimized in patients with RA leading to more effectiveness of treatment outcomes.

**Supplementary Information:**

The online version contains supplementary material available at 10.1186/s12913-021-07459-0.

## Background

Rheumatoid Arthritis (RA) is a systematic autoimmune condition, presently requiring long-term pharmacological treatment with disease-modifying antirheumatic drugs (DMARDs). Pharmacological treatment, as early as possible, is essential in preventing structural damage of joints and improving functional outcomes and improved quality of life for patients [1, 2]. Medication non-adherence is one of the most recognized factors in RA causing treatment failure, leading to increased disease activity, lower rates of remission and disappointing patient-reported outcomes [3–6]. Adherence rates in RA seem highly variable with a reported range from 30 to 99%, depending on population studied, definition of adherence used and which method of measurement is used [7, 8].

In order to improve medication adherence in patients with RA, identification of barriers and facilitators towards adherence is necessary. A recent study has identified a list of barriers and facilitators that patients experience towards their DMARDs use, such as: insufficient cognitive, communicative, or physical skills to understand and/or administer medication, lack of daily routine and experiencing side effects [9]. The individual patient’s barriers and facilitators regarding DMARDs can be discussed with the HCP to provide the room to correct possible misunderstanding, dysfunctional attributions or a lack of knowledge.

According to the World Health Organization (WHO) in 2003, adherence is a multidimensional phenomenon determined by the interplay of five sets of factors of which patient-related factors are just one determinant (World Health Organization, 2003). Also condition-related factors, therapy-related factors, healthcare team- and system-related factors, and social and economic factors are determinants for (non)adherence. This emphasizes that healthcare system factors, such as poor drug supply, unclear information about drug administration but also poor HCP-patient communication may have a negative impact on adherence. The quality of communication improves when HCPs tailor their information to the individual patient’s needs [10]. Better quality of communication might lead to improved adherence to recommendations and outcomes [11]. HCPs need to be aware of the current needs of their individual patient and adapt their communication accordingly [12, 13]. A mismatch of the individual patient’s barriers and facilitators with the perception of the attending HCP, might negatively influence treatment adherence and consequently reduce efficacy and outcomes [14–16]. As HCPs play an important role in RA management, it is important to explore their insights with regard to medication adherence in order to detect possible mismatches with the patients’ perspectives of medication use [17, 18]. However, research about HCPs’ perspectives on factors that influence medication non-adherence is scarce [19, 20]. A systematic review and meta-analysis of interventions improving medication adherence showed that interventions focussing on HCPs, significantly improved adherence among patients with chronic conditions [21]. Therefore, knowledge of HCPs’ perceptions on the barriers and facilitators regarding medication use of their patients, will help to bridge the different perceptions of both patient and attending HCP and potentially benefit adherence. This study aimed to identify the perceptions of HCPs of the barriers and facilitators to DMARD use in patients with RA.

## Methods

### Study design

Semi structured in-depth interviews were held with HCPs involved in the treatment of patients with RA. The Consolidated Criteria for Reporting Qualitative Research (COREQ) checklist was used to ensure complete and transparent reporting [22]. Eligible HCPs were rheumatologists, nurses and pharmacists, experienced in the care for patients with RA. Purposive sampling was used to obtain the perspectives of HCPs with varying professions, sex, age and regions to increase transferability.

### Interviews

Based on an adjusted Theoretical Domains Framework (TDF) [23] an interview guide was developed by the researchers to address the perceptions of HCPs on potential barriers and facilitators to DMARDs that influence patients’ adherence (see Table 1). Questions were formulated reflecting the following 11 domains: knowledge, emotions, attention/memory, decision making, social influences, beliefs about capabilities, beliefs about consequences, motivation and goals, goal conflict, environmental context and resources, skills. The interview guide was pilot tested among three HCPs. After the pilot test, no changes to the interview guide were made. Therefore, the data obtained in the pilot test were used for the final study and data analysis. All interviews were conducted by the first female author (MV) who was fully trained and experienced in qualitative interviewing. The interviews were conducted between April and September 2019. A summary of each interview (member check) was sent to each HCP to ensure their views and beliefs have been interpreted correctly by the researcher.

**Table 1:** Interview guide

DOMAIN	INTERVIEW QUESTIONS
Knowledge	1. What kind of information do patients need to have in order to take the medication as prescribed by the physician?
Emotions	2. Which emotions can be evoked in patients by the use of the medication?
Attention, memory	3a. Do you think patients are preoccupied with the medication? Can you elaborate?
	3b. Do you think patients sometimes, on purpose or accidently, forget their medication? Can you elaborate?
Decision process	3c. Do you think that patients are sufficiently involved in the decision-making process surrounding their medication? Can you elaborate?
Social influences	4. In which way do HCPs play a role in the use of the medication, other than prescribing them? Can you elaborate?
	5a. Do you think that family and/or friends of patients play a role in the use of the medication (e.g. taking or not taking the medication as prescribed)? Can you elaborate?
	5b. Do you think that the work environment (colleagues or supervisor) can influence the use of medication? Can you elaborate?
Beliefs about capabilities	6. In which situation(s) is it difficult for patients to use the medication as prescribed?
Beliefs about consequences	7. Which beliefs do patients have about the consequences of (not) taking the medication as prescribed by the physician?
Motivation and goals	8a. Which personal goals (or personal motivations) could be important for patients to use the medication as prescribed? Can you elaborate?
	8b. Which personal goals (or personal motivations) could be important for patients to *not* use the medication as prescribed? Can you elaborate?
Goal conflict	9a. What do patients consider helpful to use the medication as prescribed in daily life?
	9b. What would patients consider as barriers in daily life that hamper the use of their medication as prescribed?
Environmental context and resources	10. Do you think that the ordering, retrieving, delivery, prescribing and/or reimbursement sometimes influence patients’ use of medication as prescribed? Can you elaborate?
Skills	11. What is important for patients with RA to be able (physically as well as mentally) to use the medication as prescribed?
	12. Are there other issues important for patients that can influence their medication use?

### Recruitment and data collection

Purposive sampling was used to obtain the perspectives of healthcare professionals with varying jobs, sex, regions, age and work experiences to increase transferability. No relationship was established between the interviewer and the participants prior to the study. Participants were invited by e-mail, followed up by a phone call. They were interviewed in their own environment at a time and place of their convenience. Written informed consent to participate as well as to record the interview was obtained prior to the start of the interview. The interviews were audio-recorded and field notes were made during the interviews. Each interview was scheduled for 60 minutes. Data saturation determined the number of interviews. Intermediate analyses were conducted by two researchers (JV, MV) to decide whether new themes had surfaced. Relevant quotes of the participants were selected and translated to support the findings.

### Measures

A demographic questionnaire was completed before the start of the interview (age, sex, region, work experience in years, working in Academic/peripheral hospital).

### Data analyses

All interview-tapes were transcribed verbatim by a professional agency. The qualitative data analysis software MAXQDA 2018 (MAXQDA, 1989–2018) was used by two independent researchers (JV and MV) to code meaningful fragments in the text, conform the analysis method described by Boeije [24]. This method uses a structured way of coding [25], the principles of constant comparison [26], and in this study, an inductive thematic analysis. This method was chosen because it facilitates a theoretically flexible approach to the coding of these data. Applying this method to this study implied that the researchers interpreted the data with theoretical sensitivity to the adjusted domains of the TDF framework. Before starting the coding process, the transcripts were read and reread for familiarization with the data. The coding was done in 3 steps: open, axial and selective coding. Open coding is the part of the analysis concerned with identifying, naming, categorizing and describing phenomena found in the transcripts of the HCPs that were interviewed. These open codes were categorized (axial coding). From these axial codes the core themes and interrelatedness between themes were identified (selective coding). To support the coding process, field notes made during the interviews were used. In addition, the researchers made reflective notes about their thoughts and views regarding the identification of themes. Data collection and data analysis alternated in a cyclic process. Throughout this process, 3 authors (MV, BvdB and JV) continuously and repetitively reflected on, compared, discussed, and adjusted the coding in order to carefully determine the number and wording of themes in an iterative design (to ensure confirmability) until consensus was reached (Boeije, 2009).

## Results

Fourteen interviews (duration 45–90 min) were held at the hospital and one interview at HCP’s home. In Table 2 the participants’ characteristics are displayed. Twelve (80%) participants were female and the mean age was 47.4 (range 31–63) years. The mean in employed years was 13.1 (range 3–25) years. Four participants worked in general hospitals, three participants in university hospitals whereas eight participants had work experience in both. Data collection ended after 15 interviews as saturation had been reached after 13 interviews (no new information emerged during two consecutive interviews) [27].

**Table 2:** Participants Characteristics

Characteristic	Rheumatologist ***N*** = 5	Rheumatology nurse ***N*** = 5	Pharmacist ***N*** = 5
Mean Age (*SD*)	54.60 (*SD* = 7.30)	52.60 (*SD* = 7.27)	35.00 (*SD* = 4.64)
Sex (*F:M*)	3:2	5:0	4:1
Working experience in mean years (*SD*)	18.20 (*SD* = 5.36)	12.00 (*SD* = 5.66)	9.20 (*SD* = 4.82)
Region in the Netherlands (n)	West (2), East (1), South (2)	West (3), East (2), South (0)	West (2), East (0), South (3)
Hospital (n)	Academic (2), Peripheral (0), Academic/Peripheral (3)	Academic (0), Peripheral (2), Academic/Peripheral (3)	Academic (1), Peripheral (2), Academic/Peripheral (2)

*SD* Standard deviation, *F* Female, *M* Male

Facilitating and hampering factors for medication adherence are listed in Table 3. To present these results in a structured way and thereby enhancing readability, the identified factors were presented according to the taxonomy of medication adherence of the WHO (World Health Organization, 2003). The WHO describes five sets of factors with causes for decreased adherence: Patient-related factors, Condition-related factors, Therapy-related factors, Healthcare team and system-related factors, and Social and economic factors. All identified factors by the HCPs could be placed under these five sets of factors.

**Table 3:** Adherence factors to medication use according to healthcare professionals in this study

Categories	Facilitators	Barriers
Patient-related	Self-managing medication regimen such as: Embedding medication intake in daily routine; Tapering/dosing/discontinuing temporarily; Managing medication stock at home	Experiencing redefinition of identity
	Personality traits such as openness and conscientiousness	Feeling resistance to become dependent on (lifelong) medication
	Pursuing a solution-oriented approach	Denying the existence of condition
	Pursuing discipline in medication taking	Experiencing inner conflict of medication necessity versus concerns
	Coping with insecurity how future/condition will unfold, having a realistic expectation	Lacking confidence and/or not feeling safe enough to talk about e.g. (non) adherence
	Maintaining autonomy and improving quality of life	Having trouble to understand instruction inserts
	Being able to take care of family	Not believing in prescribed treatment, preferring traditional medication
	Being able to open medication bottles/packages and administer injections/oral medications	Not believing that medication will always be available
	Using aids to remember/motivate adherence	Not believing that condition needs (lifelong) medication
	Believing in positive treatment effect	Believing that the condition can be cured
	Experiencing positive emotions evoked by (positive experience of) medication	Attributing decreased treatment effect due to non-medical switch
		Experiencing negative emotions that are evoked by using medication for a chronic condition
Therapy-related	Experiencing a short-term onset of medication effectiveness	Experiencing side effects
	Experiencing positive treatment effect	Dreading possible interactions (medications, alcohol)
	Employing a dose-reducing strategy	Being confronted with changed appearance of medication
	Aligning patient preferences for medication administration	Perceiving information overload
		Dealing with complexity of instruction inserts
Condition-related	Experiencing high level of disease activity	Perceiving treatment as redundant without a definite diagnosis
		Experiencing a poor general health status e.g. suffering comorbidities (poly pharmacy)
Healthcare team and system-related	Providing tailored information frequently	Imposing limited consultation time to discuss choice of medication
	Discussing reasons for non-adherence before and during therapy	Lacking reimbursement medication
	HCPs are more accessible for patients (by different channels)	
	Offering optimal service logistics medication	
	Creating bond/empathy/trust with patient	
	Physician’s positive attitude on medication use	
	Initiating shared decision-making process by HCPs	
Social and Economic	Receiving social and work-related support	Travelling
		Nature of work hindering medication use (shifts)
		Perceiving negative impact of social media/internet
		Perceiving negative experiences/stories from others about medication
		Interference in medication use because of religious or cultural customs

Some factors reported by the HCPs, reflected opposite pools; for instance, short term versus long term onset of effectiveness, inner conflict of medication necessity versus concerns, and showing an open attitude versus lacking confidence and/or not feeling safe enough to talk about e.g. (non) adherence). Other mentioned factors by the HCPs were interrelated: for instance, embedding medication intake in daily routine and travelling or irregular work shifts, having trouble to understand instruction inserts and dealing with complexity of instruction inserts, personality traits such as openness and conscientiousness (e.g. discipline in medication use) and pursuing a solution oriented approach, and creating bond/empathy/trust with patient and initiating shared decision making process by HCPs.

### Patient-related factors

#### Facilitating factors to medication adherence

Patients’ strong self-management skills, such as being able to manage medication stock at home, or being able to taper, dose or temporarily discontinue medication on one’s own account because of holidays or social events, facilitated medication adherence, according to the HCPs.

Rheumatology nurse (51 years, female): *“Some patients tell us that they have not taken the medication for three months, or that they taper the medication, or that they changed the prescribed dose on their own account ( …*) *and that they choose not to mention this (to their HCPs). If you have to take your medication each week, year in and year out, you will probably think: “It is going so well, why not take a tablet less?”*

To be able to embed medication use in daily routine was stated by most HCPs as very helpful to patients. HCPs mentioned that for some patients this could be a challenge e.g. when the patient works in irregular shifts.

Pharmacist (39 years, male): “*So we accompany someone by the start of using that medicine as best as he/she can. As best as possible in his or her daily life. That is also our great challenge. I always say to our pharmacist assistant: “There is really only one thing you have to do right at the desk and that is find out what someone’s daily routine is and respond to it“. And, ‘mapping expectations’: Finding out how a person lives a life when it comes to the use of medicines and then thinking along from: “how can I make sure that someone is fully capable of doing so (medication use as prescribed).”*

Patient’s intrinsic motivation to use medication as prescribed was considered highly valuable. According to many HCPs “wanting life back as before” motivated patients to adhere to medication. This motivation for medication adherence would help the patient to set and accomplish realistic work, leisure and family related activities and goals, and ultimately to maintain autonomy and (improve) health-related quality of life. Reasons to be adherent were, according to all HCPs, the experienced treatment effect, ultimately leading to often improvement of quality of life. Another reason which was mentioned frequently was that patients are motivated to use their medication as prescribed, in order to be able to take care of family. This could be in the role of caregiver (taking care of disabled parents or spouse) or in taking care of the children.

HCPs also mentioned that conscientious patients (e.g. having discipline in medication taking, having the patience to wait for a treatment effect), patients with an open attitude that would enhance communication with the HCPs, and patients who are able to successfully cope with insecurity between how their condition will unfold in the future, were considered to better adhere to their medication regime.

HCPs stated that in their experience, besides positive emotions and feelings that could arise from taking medication and experiencing a treatment effect, patients’ belief that treatment effects would outweigh side effects, the belief that the use of lifelong medication would improve quality of life, and realistic expectation of how the treatment would affect the condition, contributed to medication adherence.

Practical issues that helped medication use were mentioned by the HCPs as well. For instance to be able to open medication bottles or packages, be able to swallow tablets or to administer injections. Providing aids, such as reminders and pill boxes, were considered as helpful to medication use in case of non-intentional non-adherence.

#### Barriers to medication adherence

HCPs indicated that patient’s negative emotions such as aversion, sadness, anger, and problems with self-image were likely to hamper medication adherence. Problems were described with poorer self-image, describing that patients felt that the use of medication defined them as patients, since living with a lifelong condition meant living with lifelong medication. Patients did not want to be associated with this image all the time. To resist these identity redefinition processes, sometimes denial of the condition was observed and resulted in non-adherent behaviour.

The HCPs thought that many patients did not feel free and safe enough to talk about personal drug-related issues related to their condition, and this affected adherent medication behaviour. For instance, patients’ wish to have children that may require a change in medication strategy. According to the HCPs, many patients still feel intimidated by the status of the treating physician, hampering initiating a conversation about changing the medication regimen.

Pharmacist (31 years, female): “*I think that patients admit non-adherence easier to us than to the rheumatologist. They say sometimes: “I am not always taking my medication”, or: “While on holiday, I have not used my medication for several weeks”, or: “When I feel fine, I take less medication than agreed with the rheumatologist”.”*

HCPs expressed that patients’ beliefs and misconceptions about the (long-term) side-effects could hamper medication adherence, as patients can be conflicted about the need for medication versus the concerns they (might) have about the medication they take.

Some HCPs mentioned that some patients mistakenly believed that the condition could be cured by taking medication or that the rheumatic condition would not require (life-long) medication. Other patients refused medication as they were unsure about the availability of medication in the future and they fear to be dependent on one particular medication. Some patients do not believe in the prescribed medication and they prefer traditional medication instead. Some HCPs stated that patients have difficulties to understand the instruction inserts of their medications. One HCP mentioned that after a non-medical switch (prescribing a biosimilar after the prescription of a biological), the patient attributed the decreased treatment effect to the biosimilar, and in turn hampered willingness to take the medication.

Rheumatologist (45 years, female): *“We put a lot of people on biosimilars at the time. We’ve talked to our patients about that at length. It’s the same drug. Well, yes, you’re now experiencing that it works less. It can, it may, indeed, that it works less. The effect of the drug is becoming less and that could have happened with that other drug as well. I don’t know if we should give people a lot of information about that right now, if it’s really the same drug in the end?”*

### Therapy-related factors

#### Facilitating factors to medication adherence

According to the HCPs, a short term to onset of effect of the medication was one of the therapy-related factors facilitating adherence. An experienced positive treatment effect contributed to better adherence as well. HCPs considered the option for tapering medication as a motivation for the patient in order to adhere to the treatment plan. Explaining and discussing the choice in how medication can be administered, especially at the start or when a change is required in the medical treatment, was also considered important to adherence to take away fear and anxiety, according to the HCPs.

Rheumatology nurse (51 years, female): “*What do you like more: once a week an injection – you have to do that yourself – or an IV ... I have a number of patients who say, “I think it’s – actually – fine. A few hours here in that chair, just reading my paper. That’s really a relaxing moment”.”*

#### Barriers to medication adherence

The HCPs reported that the barrier most often mentioned by patients, was experiencing side effects of their medication use. Dreading possible interactions, such as using concomitant medications that patients fear to be contraindicated, or using medication in combination with alcohol or certain drugs, were also given as reasons that patients may be less adherent to their prescribed medication for their rheumatic condition. Another presumed barrier for adherence according to the HCPs, is the change of the appearance of medication, such as colour or packaging boxes. With regard to therapy related information for patients, many instruction inserts are sometimes too complex and too long, risking misinterpretations and information overload. According to the HCPs, communication is key to prevent or overcome these potential barriers.

### Condition-related factors

#### A facilitating factor to medication adherence

A condition-related factor that may positively influence patients’ medication use according to some HCPs, was the level of disease activity. When the disease activity was high, patients were more willing to use their medication as prescribed.

#### Barriers to medication adherence

Most HCPs stated that a poor general health status, such as health problems beside the rheumatic condition, can hamper medication use. For instance, comorbidities that require poly pharmacy, or temporarily health issues, such as having a cold resulting in less wellbeing. They felt that without proper informative communication about the necessity of different medications for different conditions, patients are less inclined to use these. The absence of a definite diagnosis was mentioned as a barrier that would make the patient reluctant to follow a proposed treatment plan.

Rheumatologist (50 years, male): *“I say to my patient; “Of course, it doesn’t always matter if you have symptoms, to come to a diagnosis ( …*). *The symptoms are the complaints and you can have them without a diagnosis. Rheumatism is all about medicine for the first year. (..) The second year is very often about “it lands”, that you feel that your body is not like before. That it is no longer real to demand that you will be as before. And that doesn’t come until the second year. And then, of course, you still have all the stages of life”. And that’s what I mean: you have to look a little bit at what stage someone is in (with regard to a prescribed medication regimen).”*

### Healthcare team and system-related factors

#### Facilitating factors to medication adherence

Providing the same information by different HCPs, tailored to the patient and repeated when necessary, would benefit the patient according to the HCPs. Especially after diagnosis when medication is commenced or when a change in medication strategy is required, clarity and information about treatment could help with the acceptance of a lifelong condition and subsequently of life-long medication use.

Some HCPs mentioned that barriers to non-adherence should be discussed before and during therapy, since non-disclosure for non-adherent behaviour can lead to unnecessary decisions for treatment change (other medication or different dose). They stated that creating easier access for patients to the HCP when needed, for instance through e-mail, would most likely facilitate adherence.

Rheumatology nurse (51 years, female): *“We (colleagues) have already defined the important topics that need to be addressed with the patients, before they come for their first visit. One of these points is the need for probably long-time use of medication. We also explain that we have a schedule to start, use and maybe taper the medication with the focus on best results with as less medication as possible. But, yes, it is a shock for them … I understand. When you have to use medication year in, year out, that is sad information. It must always “land”.”*

Some HCPs mentioned that improvements of the service level with regard to medication logistics could facilitate medication use, e.g. delivering medication at home, an easy way to get a refill at the pharmacy and patient-friendly packaging of medication.

A few HCPs stated that adherence to medication can sometimes be attributed to the attitude of the physician towards medication use. According to these HCPs, a strong positive attitude with regard to certain medication can influence the willingness of patients to use this medication as prescribed.

The HCPs also expressed that creating a bond with the patients, specifically in chronic conditions, may lead to more mutual empathy and trust and subsequently to more room for discussion, providing and receiving information about more sensitive topics such as barriers to adherence to medication.

Rheumatologist (55 years, female): *“For me it is really important to feel whether patients are taking their medication or not, because only then I can judge whether they are effective or not. Nothing can be as bad as prescribing a higher dose when the condition is progressing, when ultimately it turns out, no matter what the cause is, that the patient was non-adherent (for a while). It would be crazy to change the dose in this case. It is not effective”.*

The HCPs stated that for most patients, shared decision-making is essential for adherence to a treatment plan. In their view, the HCP should initiate this process, in collaboration with the patient and if applicable, the rheumatology nurse.

Rheumatologist (50 years, male)*: “I think that doctors are still trained in the way of ‘thinking in lists’. Symptoms lead to a diagnosis. This diagnosis leads to a treatment plan and the patient has to conform to it. The involvement of the patient is mainly in the implementation phase. I think it is only in recent years that we have come to realise that this is a very limited model. (..) If you ask someone to perform, it only works well, if that patient knows why they should do so and is better informed about it. I think we know much better by now, that the quality of care and the effect of care is really only good, if someone is involved in decision-making at all stages and thinking along in the various steps of the treatment plan.”*

Subsequently, the opposite was highlighted as well, many HCPs felt that patients were often not given a choice between eligible medication strategies, partly because some HCPs do not want to take the patient’s decision into account, or because of the prescribed protocols that need to be followed, leaving no room for shared decision-making.

Pharmacist (31 years, female)*: “We think patients will start using their medicines better, if they feel that they can decide in: “What are we going to do about it now?” As caregivers, we are inclined to think, “This is our protocol. ( … )This is step one of the protocol and so we are going to do it this way.” I think it’s shifting a little bit, but how is it going to be a joint decision? I think that’s kind of hard for doctors, of course, to have a protocol on the one hand, (…) whereas that patient might have something else in mind. It is a difficult dilemma”.*

#### Barriers to medication adherence

Factors that were mentioned by the HCPs as barriers in medication use, were, e.g., limited consultation time imposed by regulatory authorities: this was considered too short to discuss extensively the medication strategy. Some HCPs stated that costs of medication and (lack of) reimbursement of medication are sometimes reasons for patients not to collect their medication at the pharmacy, leading to non-adherence. This was remarkably mostly mentioned about less expensive drugs, since, e.g., bDMARDS are reimbursed by insurance in the Netherlands.

Rheumatology nurse (55 years, female): “*Then they’re not going to use the drugs that aren’t being reimbursed anymore, and then they’re going to be in a lot of pain. Then they actually want other painkillers, which are reimbursed. But those often have other side effects”.*

HCPs reported that another reason for patients to stop their prescribed medication was awareness about the high costs for medication such as biologics. Patients felt that this money could be spend more useful e.g. for environmental friendly projects or to development projects in poor countries.

Rheumatologist (45 years, female): “*Another great example – I think – patients didn’t want to use biologicals anymore, because they are so expensive. She thought that the money should go to a project in India. That’s one. There’s been another one, who also said, “This is too expensive. We, as a society, should not want this.” Yes, she just didn’t want to. Really, they said, “From an economic point of view, I just don’t want this”.”*

One HCP felt that their considerations about the choice of medication with regard to costs, can be shared and explained to patients in order to gain understanding resulting to improved adherent behaviour.

Rheumatologist (63 years, female): *“What I also mean here: as a doctor, you take the costs into account, so why do we want to keep that away from our patients so constrainedly. In fact, I think we can be transparent about it (that sometimes costs also determines our choice (room for options)).... As long as we can justify that preference in relation to the health gain, it’s fine, isn’t it?”*

### Social and economic factors

#### Facilitating factors to medication adherence

According to some HCPs, most patients rely on social support with regard to their medication use. This social support not only includes support from family and friends, but support from their work environment as well, such as support from the employer and colleagues. This support can be the understanding, empathy, or help with physical or cognitive challenges, such as dividing work load and offering adjusted working hours (support at work) or assisting in administering the medication or reminding medication intake (social support). As opposite, fear for loss of career opportunities in the work environment, could lead to non-disclosure of condition to the employer and colleagues and to non-adherent behaviour.

Rheumatology nurse (63 years, female): *“Yes, people still think: “Rheumatism? Oh, those are dropouts, contract but not renew.”*

#### Barriers to medication adherence

A factor that was mentioned by most HCPs as a social and economic barrier to patients’ medication adherence, was travelling, due to difficulties with scheduling medication intake, requirements for certain contraindicated vaccinations and difficulties with medication storage. According to the HCPs, discussing these inconveniences with regard to medication use with the patients can sometimes lead to simple solutions.

Rheumatology nurse (63 years, female): *“In case of travelling and you need to inject during your holidays, we often advise (after agreement among our team): “You know, just leave your injection for a week, because the risk of infection in terms of storage and temperature is higher than skipping one time your medication”.”*

Furthermore, the HCPs mentioned that barriers such as the impact of social media, or stories in newspapers or online about negative medication experiences as well as stories about negative experiences from peers about medication, may trigger non-adherent behaviour. For some patient groups, religious reasons can also affect medication use, such as the Ramadan (not allowing believers to consume food or drinks during daytime, often required in combination with medication).

## Discussion

In this study, HCPs involved in rheumatology care reported multiple factors they perceive to influence medication adherence in patients with RA.

Besides therapy-related factors, such as (onset of) medication effectiveness and medication side-effects, the largest variety was found within patient-related factors and reflected patients’ beliefs, ways of coping, and (self-management) skills toward medication and their condition. In addition, factors related to the condition (e.g., level of disease activity), healthcare team and system (e.g., trust in HCP), and social and economic context (e.g. social and work-related support) were reported.

Two previous studies reported the insights of rheumatologists into medication adherence in patients with immune-mediated inflammatory conditions [17, 18]. Differences between findings in this study and findings of previous research predominantly reflect cultural and health system/contextual factors. In contrast to the findings of Heidari et al., [18], access to, and availability of medication was not mentioned as a barrier to adherence by the HCPs in our study. In the Netherlands, all patients have medical insurance and costs of (expensive) medications are fully reimbursed. Access to healthcare professionals and pharmacies are firmly embedded in the Dutch healthcare system. Furthermore, in contrast to Ammoury et al., [17] the interviewed HCPs in our study did not report on certain, more or less fixed, patient-related factors such as disease duration, sex, age and smoking as factors for medication adherence. An explanation might be that according to the interviewed HCPs, patients will probably not give their sex as barrier to medication use, since they may not be aware of a possible association between these factors and (non) adherence. Furthermore, in contrast to Heidari et al., [18], HCPs in this study and patients in our previous study [9] mentioned that support, both in a professional and in a social environment, is important with regard to medication use. Besides these differences, most factors identified in this study are in line with the majority of barriers and facilitators reported in previous mentioned studies.

Most barriers and facilitators of medication adherence mentioned by the HCPs in our study were patient-related. However, this conclusion should be interpreted with care: the interview guide used in this study may have influenced the variety in the reported factors, since most questions focussed on patient-related issues, such as e.g. beliefs, emotions, motivation and goals. It is therefore essential to be aware of the fact that the frequency and variety of mentioned factors by the HCPs in this study yields no indication of the impact of these factors for medication adherence. This requires a different study design, such as a maximum difference scaling exercise [28]. Furthermore, for the interpretation of barriers and facilitators that influence patients’ adherence reported by the HCPs, it is important to take contextual factors (e.g. socioeconomic situation of countries, healthcare system) into account when optimizing medication adherence strategies involving patients and HCPs.

Notably, the majority of barriers and facilitators to DMARD use reported by the HCPs in this study are consistent with those reported by patients in previous studies [9, 29–31], and with the results of a recent systematic review that summarizes factors for medication adherence across several patient populations, showing that most factors contributing to medication adherence were patient-related [32]. This suggests that Dutch HCPs seem to be well aware of barriers and facilitators that may hinder or help patients with regard to their DMARD use resulting in (non) adherent behaviour.

### Clinical relevance

As HCPs seem to be well aware of the potential barriers interfering with the use of medication by patients, the question arises why these potential barriers to medication use are often not the topic in consultations, and even described as “conspiracy of silence” [33]. Especially given the fact that most modifiable barriers can be addressed in patient education and self-management interventions, such as having trouble to understand instruction inserts, not believing in (the need for lifelong) prescribed medication, not believing medication will always be available, or believing that the condition can be cured [34]. Furthermore, effective (e-health) interventions that are relatively easy to use, can help to address patients’ health literacy, patients’ self-efficacy to manage their condition and may help to overcome the limited consultation time of physicians by offering other supportive ways for patients to discuss their barriers for medication adherence [35]. As for interpersonal factors, a trusting bond between HCP and patient, to be able to discuss adherence barriers and facilitators and providing tailored information in all phases of the treatment process (e.g. before treatment initiation, in the treatment initiation phase and in the treatment persistence phase) were mentioned as facilitating medication adherence. Several studies report that the relationship between healthcare professional and patient is key to enhance adherence and health outcomes [8, 36]. In a recent study of Roodenrijs et al., the presence of adverse events was prioritized as the most important adherence barrier and a good relationship with the HCP was prioritized as the most important adherence facilitator [37]. Although in this study, we have explored, but not prioritized, which barriers and facilitators are important for medication adherence according to HCPs, findings can be interpreted as comparable since the experience of side effects and creating a bond/empathy/trust with the patient, were factors that were mentioned by most HCPs. A good relationship with the HCP, described in the study of Nota et al., involves themes such as mutual respect between HCP and patient, trust and an open style of communication [38].

HCPs endorsed the view that they should create the conditions for shared decision-making (SDM) during their encounters with the patient to create a common playing field with the patient while discussing the pros and cons of medication and medication adherence in different stages of the condition. Although most RA patients prefer SDM, their preference may vary according to the situation they are in (type of treatment and severity of complaints) and the extent to which they experience barriers in getting more actively involved [38]. Unawareness of having a choice is still a major barrier for patient participation. The HCP has an important role as facilitator in enhancing patient participation by raising awareness and offering options, but implementing SDM is a shared responsibility; all parties need to be involved and educated [38].

### Future research

In a previous maximum difference scaling exercise, patients identified the most important barriers and facilitators [28]. Reducing symptoms, maintaining independency and shared decision-making were found to be the three most important contributors to medication adherence. Factors identified in the current study are in line with these latter observations, although a difference scaling exercise could add knowledge of the relative importance to the factors identified by the HCPs in this study.

### Strength and limitations

The strength of this study is that the qualitative theory-based in-depth interviews were conducted with different health professionals. This provided insight from different healthcare professionals’ perspectives into patients’ facilitators and barriers to medication use.

As limitations, the use of a predetermined interview guide based on an adjusted TDF model [9, 39, 40], could be considered as a stringent way to identify possible barriers and facilitators for medication use, however, by adding a question if there were factors that HCPs felt important to mention beside the factors already discussed, this potential limitation has been accounted for. No additional factors have been mentioned at the end of each interview.

In addition, the sample size of this study was limited to 15 healthcare professionals. Yet, saturation was reached in the 13th interview. This is in line with the findings of a review study by Guest et al. [27] into saturation and variability. They found that saturation can occur within 12 interviews and elements of key themes can already be presented after six interviews. Data collection and analyses were alternated to ensure that saturation could properly be established.

Furthermore, this study was conducted in one healthcare system of a single European country, which may limit the transferability of the findings, particularly to non-Western countries.

## Conclusions

In summary, multiple barriers and facilitators have been identified that influence patients’ medication adherence according to their physicians, nurses and pharmacists. Largest variation in barriers and facilitators were patient-related. According to the outcome of this study, Dutch HCPs are aware of the reasons for patient’s non-adherence, therefore adherence should be a topic in consultations between HCP and patient. Our findings can be the base for the development of (digital) interventions and/or health services to increase medication adherence among patients using disease modifying anti-rheumatic drugs. Most mentioned barriers in this study, such as personal (e.g. patients’ beliefs and knowledge) and interpersonal factors (e.g. the quality of the patient-provider interaction) are potential modifiable and can be addressed to enhance medication adherence.

## Supplementary Information

**Additional file 1.**

## Data Availability

A unique study code has been appointed to each health professional. Thereby, the data from the questionnaire and transcripts are anonymous and can only be tracked back by an identification log. This identification log can only be accessed by the primary researcher (MV). Transcripts of interviews (anonymous format), and signed informed consent forms have been stored on a secure network drive for 20 years. Digital audio files have been stored on a secure network drive in an anonymous format for 10 years. Study data were entered and analysed in the software MAXQDA® 11 (VERBI software GmBH, Germany). The anonymised data generated and/or analysed during the current study are available from the corresponding author on reasonable request.

## Abbreviations

DMARDs: Disease-Modifying Anti-Rheumatic Drugs
TDF: Theoretical Domains Framework
RA: Rheumatoid Arthritis
HCPs: Healthcare Professionals
WHO: World Health Organisation
COREQ: Consolidated Criteria for Reporting Qualitative Research
SDM: Shared Decision Making
